# Vanishing Polarizability of Dark Excitons in WSe_2_: Implications for Noise-Resilient Quantum States

**DOI:** 10.1021/acs.nanolett.6c01047

**Published:** 2026-04-27

**Authors:** Ali Soleymani, Qiaohui Zhou, Keyuan Bai, Fei Wang, Kenji Watanabe, Takashi Taniguchi, Jiang Wei, Xin Lu

**Affiliations:** † Department of Physics and Engineering Physics, 5783 Tulane University, New Orleans, Louisiana 70118, United States; ‡ Research Center for Electronic and Optical Materials, 52747 National Institute for Materials Science, 1-1 Namiki, Tsukuba 305-0044, Japan; § Research Center for Materials Nanoarchitectonics, 52747 National Institute for Materials Science, 1-1 Namiki, Tsukuba 305-0044, Japan

**Keywords:** tungsten diselenide (WSe_2_), dark exciton, spin triplet, Stark shift, polarizability, electric field

## Abstract

The spin-triplet dark excitons in transition metal dichalcogenides, such as WS_2_ and WSe_2_, are promising for quantum information processing due to their long lifetimes and robust spin–valley states. While effectively utilizing these states requires a precise understanding of their modulation by external controls, it remains less explored compared to the bright counterparts. Here, we investigate the out-of-plane electric field dependence of the neutral dark (*D* ^0^) exciton in the dual-gated monolayer WSe_2_. Contrary to the large Stark shifts observed in interlayer excitons, we demonstrate that the *D* ^0^ exciton exhibits a vanishingly small polarizability (<6.7 × 10^–10^ D·mV^–1^). We attribute this electrical immunity to strong spatial confinement. Crucially, we show that this insensitivity extends to the magnetic response, where the *g*-factor remains robust against electric field variations. Our results establish the dark exciton as a noise-resilient building block for future spin–valley quantum information processing.

## Graphical abstract

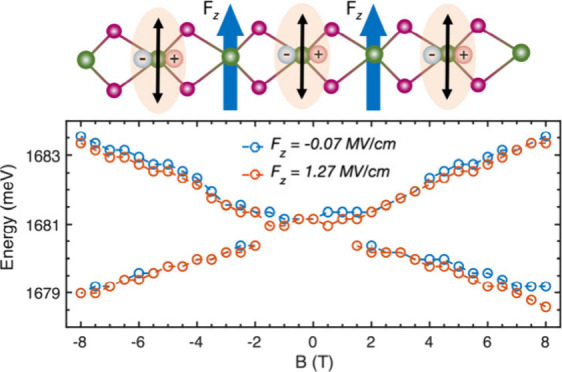

Atomically thin transition metal dichalcogenides (TMDs) have emerged as a frontier platform for nanophotonics and quantum information science, driven by their tightly bound excitons and unique spin–valley physics.^1,2^ A key advantage of TMDs is the dynamic tunability of their optical properties via external stimuli such as strain,^3,4^ dielectric,^5,6^ electrostatic doping,^7,8^ and electric field.^9–11^ In particular, electrical control has been extensively studied and shows great promise for optoelectronics,^12^ integrated photonics,^13^ and quantum information science.^14^ In contrast to electrostatic doping, which alters the excitonic species, the presence of an electric field only shifts the energy of the exciton without changing its components in the low-field limit. Electric field-modulation has been successfully demonstrated in the homo- and heterobilayers, where the interlayer exciton has a permanent dipole.^15–18^ However, this high sensitivity to electric fields comes with a trade-off: excitonic states that are easily tuned are also highly susceptible to local electric field fluctuations, which serve as a primary source of dephasing and decoherence in solid-state qubits. Therefore, identifying excitonic states that combine long radiative lifetimes with intrinsic immunity to electrical perturbations is critical for realizing robust quantum memory and coherent spin–valley interfaces.

In light of these considerations, the spin-triplet dark excitons in tungsten-based TMDs are a compelling candidate. Unlike their bright counterparts, dark excitons possess significantly longer lifetimes^19,20^ and strong interactions with lattice vibrations,^21–24^ making them attractive for long-term information storage. Previous experimental investigations revealed that the neutral bright (*X* ^0^) exciton exhibits a significantly larger in-plane polarizability,^9–11^ whereas its out-of-plane polarizability is 5 orders of magnitude smaller.^25^ Nonetheless, the electrical response of the neutral dark (*D* ^0^) exciton remains experimentally unexplored due to the difficulty in optical accessibility. Knowing the polarizability of the *D* ^0^ state is essential to determine its resilience against charge environment fluctuations. It remains an open question whether the out-of-plane oscillating *D* ^0^ exciton is highly electrically tunable like the interlayer species or whether it possesses a structural stiffness that protects it from electrical noise.

We first compare *X* ^0^ and *D* ^0^ excitons in the momentum and real spaces (Figure 1a,b). While the electron and hole have the same spin orientation in the *X* ^0^ exciton, their orientations are different in the *D* ^0^ states (shown in different colors). The transition dipole in the spin-triplet *D* ^0^ exciton points out-of-plane. Due to the mirror symmetry, emission propagates primarily in-plane, thus rendering the *D* ^0^ exciton “dark” in some earlier studies.^25–27^ The observation of the *D* ^0^ exciton typically requires a “clean” device with minimal contamination, an objective with a high numerical aperture (NA), and low temperatures. The schematic of our device is illustrated in Figure 1c, and Figure 1d shows an optical microscope image of a real device (Device 1). Although it is more complicated in fabrication, one advantage of the dual-gate geometry is the independent control of carrier density and out-of-plane electric field. The dual-gate design can further compensate for the electron tunneling effect, which may occur at high electric fields when the Fermi level in the graphene gate is above the conduction band minimum in the WSe_2_;^28^ it also adds flexibility in fine-tuning when the excitation source induces the photodoping effect.^25^

**Figure 1:**
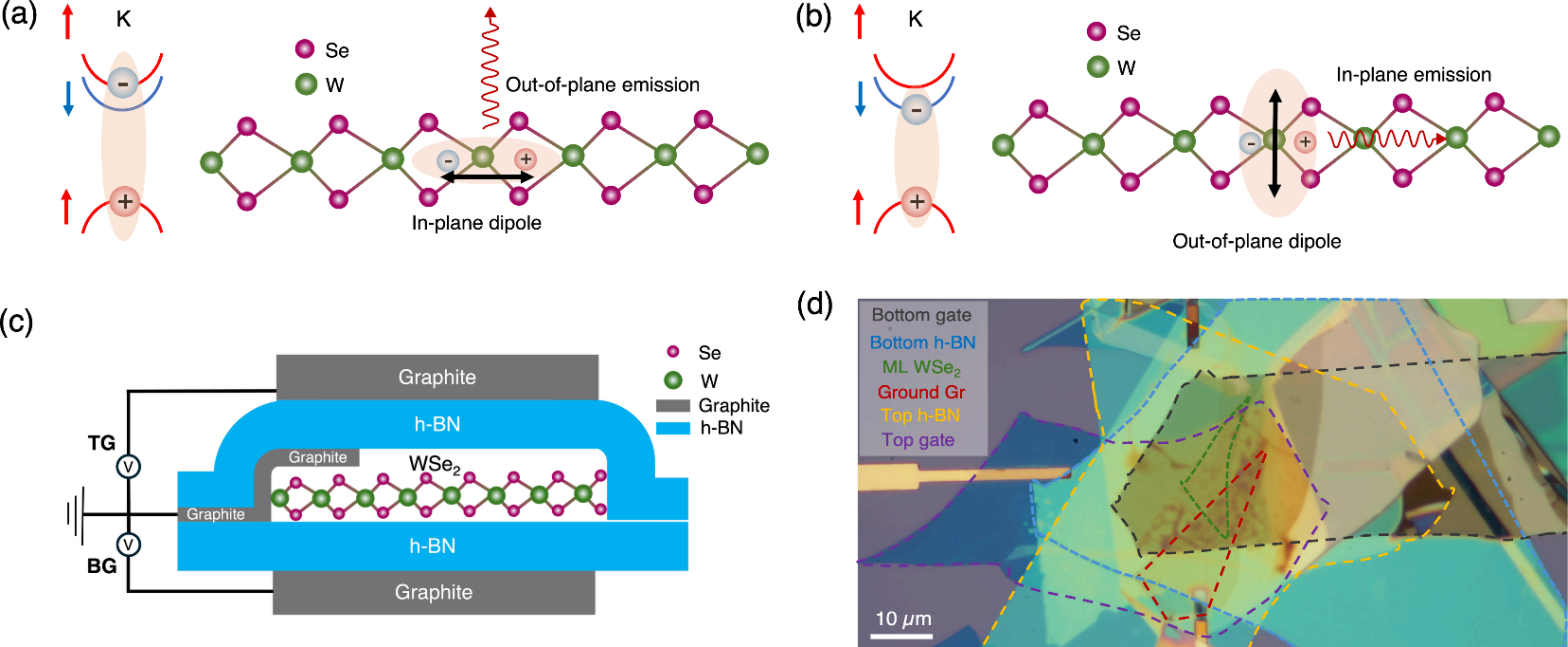
Exciton configurations and device geometry. (a, b) Comparison of the *X* ^0^ (a) and *D* ^0^ (b) excitons in the momentum space (left) and the real space (right). (c) Schematic of the dual-gated device. (d) An optical microscope image of Device 1.

To identify the *D* ^0^ exciton, we carried out gate-dependent photoluminescence (PL) measurements at ∼4 K. Figure 2a displays the cross-circularly polarized PL color plot as a function of *V*_BG_ when *V*_TG_ = 0 V (from Device 2). Our assignment of the neutral and charged bright excitons is in consistent with earlier results.^21–24^ In addition to the bright excitonic complexes, we also observed a strong peak whose energy is ∼40 meV below the *X* ^0^ peak and matches the energy of *D* ^0^. We tentatively assigned this peak to be the *D* ^0^ exciton and further noticed the emission from its phonon replica (*D*_Γ_5__^0^ in Figure 2a) to be red-shifted by ∼22 meV relative to *D* ^0^. This observation corroborates the assignment of the *D* ^0^ exciton.

**Figure 2:**
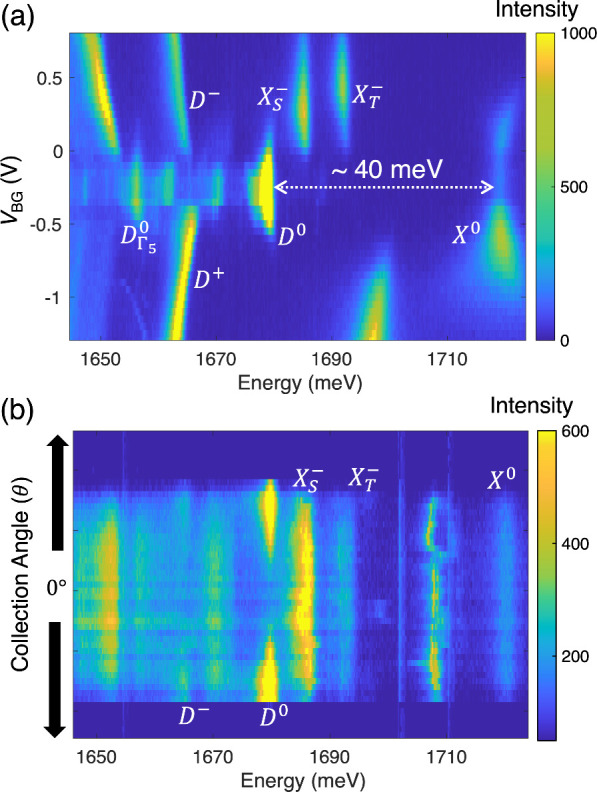
Identification of the *D* ^0^ exciton. (a) Low-temperature gate-dependent PL color plot of monolayer WSe_2_, showing the bright (*X* ^0^, *X* ^+^, *X*_*S*_^–^, *X*_*T*_^–^) and dark (*D* ^0^, *D* ^+^, *D* ^–^) excitonic states as well as the phonon replica (*D*_Γ_5__^0^). (b) Collection angle dependence of emission, which demonstrates the different emission patterns between the bright and dark excitons. The emission range of ±54° is determined by the objective (Figure S1).

We further employed back-focal-plane (BFP) imaging to confirm the physical origin of the peak. Figure 2b shows the collection angle (θ)-dependent emission from the same device (see Figure S1 for a schematic diagram). Despite dominating in the PL color plot (Figure 2a), the *D* ^0^ exciton at ∼1680 meV completely vanishes at 0°. Its emission is localized at the edges when θ is large. Different from the dark exciton, the *X* ^0^ exciton is more disperse and dimmer at the edges (the detailed pattern depends on the dielectric environment^29^). In addition to the neutral excitons, we notice that the charged excitons (*D* ^–^, *X*_*S*_^–^, and *X*_*T*_^–^) exhibit the same angle dependence as their neutral counterparts. Our BFP measurements reveal the distinct emission features between the bright and dark excitonic complexes and further supports the assignment of the dark state.

Having confirmed the *D* ^0^ excitonic peak, we proceeded to examine its dependence on the Fermi level: whether deviation from the charge neutral point would cause peak shift or broadening. As shown in Figure 2a, while carrier-doping shifts the energies of the *D* ^–^, *D* ^+^, and *X* ^+^ excitons, the charge-neutral *D* ^0^ and *X* ^0^ peaks hardly shift when the Fermi level is in the gap. In Figure 3, we extracted the *V*_TG_-dependent peak width, energy, and intensity from Device 1 when *V*_BG_ = 0 V. (The electric field is negligible in the charge-neutral regime when both gates have similar values). In order to avoid the potential pseudomagnetic field from circular excitation,^30–32^ we used linearly polarized light for this measurement (see Figure S2 for the PL color plot from Device 1). The *X* ^0^ exciton exhibits a narrow peak with a linewidth of ∼4 meV (Figure 3a), indicating that the inhomogeneous broadening is suppressed.^33^ Due to scattering with carriers, the *X* ^0^ peak is broadened with either electron- or hole-doping (n-doped or p-doped in Figure 3a), accompanied by a blueshift from the phase-space filling effect (Figure 3b). Interestingly, the *X* ^0^ exciton does not demonstrate the strongest intensity in the charge neutral regime (Figure 3c), as exchange scattering increases the emission rate when the sample is hole-doped.^24^

**Figure 3:**
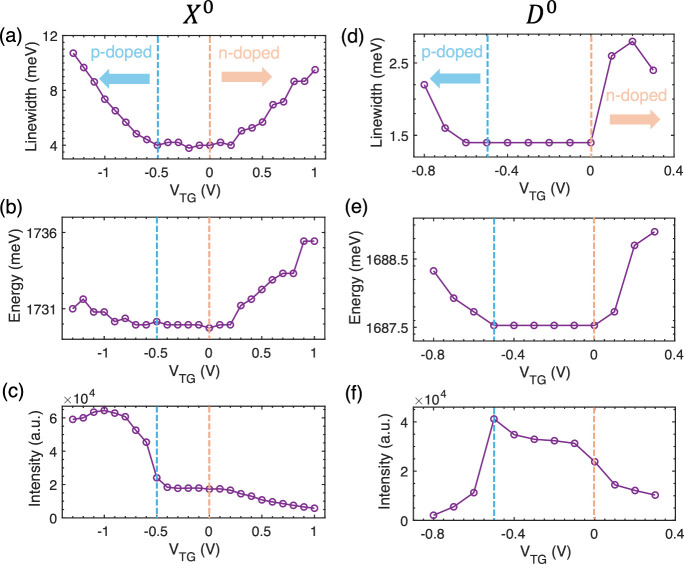
Gate-dependent peak width, energy, and integrated intensity of neutral excitons *X* ^0^ and *D* ^0^ measured from Device 1. (a–c) *V*_TG_-dependent peak linewidth (a), energy (b), and intensity (c) of the *X* ^0^ exciton. (d–f) *V*_TG_-dependent peak linewidth (d), energy (e), and intensity (f) of the *D* ^0^ exciton. The charge-neutral region lies between the dashed lines. Carrier doping (p-type or n-type) is indicated by the labeled arrows. *V*_BG_ = 0 V during the measurements.

Different from the *X* ^0^ peak, discussion on the out-of-plane *D* ^0^ exciton is not as comprehensive. Figure 3d demonstrates that the *D* ^0^ exciton exhibits an even narrower linewidth of ∼1.5 meV. Such a narrow linewidth facilitates the detection of small peak shift and broadening. Same as the *X* ^0^ peak, the *D* ^0^ exciton also broadens and blue-shifts (Figure 3e) with carrier injection. The intensity of *D* ^0^ reaches its maximum in the neutral regime (Figure 3f), as expected from a neutral exciton. We notice that the energy and width of the *D* ^0^ exciton are both stable in the entire neutral regime, which indicates that the *D* ^0^ exciton is robust against carrier screening and carrier scattering as long as the Fermi level is located within the gap.

Figure 4a shows the PL spectra of the *X* ^0^ and *D* ^0^ excitons at three different electric fields. The weak signals between 1690 and 1720 meV can be assigned to the precursors of charged excitons (similar observation by He et al.^21^). Although the charged peaks are not quenched completely, we note that the small concentration of carriers will not affect the excitonic emission, as demonstrated in Figure 3. By matching the boundary conditions, the out-of-plane electric field (*F*_*z*_) seen by the monolayer WSe_2_ is described as

$$F_{z}=\frac{V_{diff}}{d_{w}+d_{BN}\frac{\varepsilon_{w}}{\varepsilon_{BN}}}$$

where *V*_diff_ = *V*_BG_ – *V*_TG_, *d*_w_ = 0.7 nm is the thickness of the monolayer WSe_2_, and *d*_BN_ is the total thickness of the top and bottom h-BN layers (see Table S1). ε_w_ = 7.2 ^34^ and ε_BN_ = 3.76 ^35^ are the dielectric constants of WSe_2_ and h-BN, respectively. We plotted the *F*_*z*_-dependent peak width and position in Figure 4b–e. In contrast to the *X* ^0^ exciton whose width is insensitive to *F*_*z*_ (Figure 4b), the *D* ^0^ peak is broadened at high electric fields (when |*F*_*z*_| > 1 MV/cm), indicating the field-induced dephasing effect (Figure 4c). Due to the nature of in-plane dipole, the *X* ^0^ exciton is not expected to shift substantially with increasing |*F*_*z*_| (Figure 4d),^25,28^ while the out-of-plane oscillating *D* ^0^ exciton should be more sensitive. While an initial observation suggested a marginal peak shift from the *D* ^0^ peak (Figure 4e), subsequent measurements across multiple devices and cool-down cycles (Figures S3–S6) confirm that the *D* ^0^ exciton exhibits a negligible shift even when |*F*_*z*_| reaches ∼1.7 MV/cm. Given our spectrometer’s pixel resolution of ∼0.2 meV, any potential energy shift is strictly bounded by this detection limit. Using this robust upper bound, we calculate the out-of-plane polarizability (α_⊥_) via the Stark effect, $\Delta E=-\frac{1}{2}\alpha_{⊥}F^{2}$ With the Stark shift (Δ*E*) being <0.2 meV at *F*_*z*_ = 1.7 MV/cm, our result establishes that the out-of-plane polarizability of *D* ^0^ exciton is less than 6.7 × 10^–10^ D·mV^–1^ (1.38 × 10^–2^ eV·nm^2^V^–2^).

**Figure 4:**
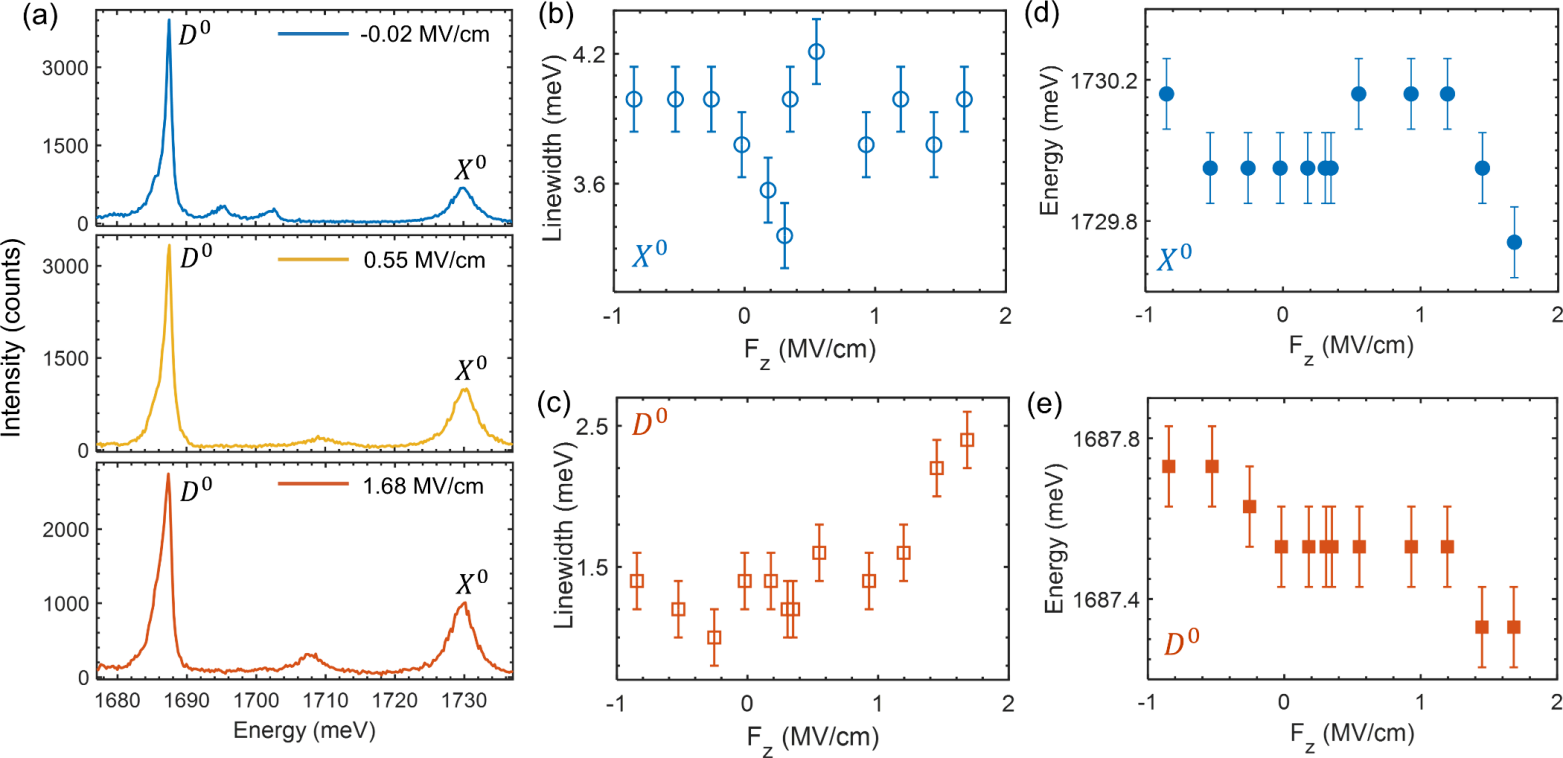
Electric field dependence of *X* ^0^ and *D* ^0^ excitons in Device 1. (a) PL spectra of *X* ^0^ and *D* ^0^ excitonic peaks at −0.02, 0.55, and 1.68 MV/cm (from top to bottom). (b, c) *F*_*z*_-dependent peak width of *X* ^0^ (b) and *D* ^0^ (c). (d, e) *F*_*z*_-dependent peak energy of *X* ^0^ (d) and *D* ^0^ (e). The data were taken at the base temperature during the first cool-down cycle. See Figure S3 for the second cool-down cycle.

Table 1 summarizes the experimentally measured out-of-plane polarizability of the *X* ^0^ exciton in TMDs, which shows that the polarizability from WSe_2_ is substantially smaller than that of other TMD materials. It is important to note that these values are derived from disparate experimental setups, encompassing different dielectric environments and temperatures. Consequently, the observed variations likely reflect a combination of intrinsic material differences and systematic variations across experimental platforms. Yet, to understand the measured values, one can start by including spatial confinement in modeling the out-of-plane polarizability. Pedersen assumed an infinite-barrier square well for electrons and holes with both barrier heights being identical. By ignoring the atomistic details and Coulomb effect, the out-of-plane polarizability is calculated to be ∼10^–9^ D·mV^–1^ (2 × 10^–2^ eV·nm^2^V^–2^), assuming that the total out-of-plane effective mass is 2 and thickness is 0.65 nm.^36^ The larger polarizabilities observed in MoS_2_ ^37,38^ and WS_2_ ^39^ could be explained by the leakage of the wave function into the barriers. The even smaller value in WSe_2_ ^25^ is attributed to the stronger Coulomb interaction in tungsten-based TMDs, which results in a larger binding energy and smaller electron–hole separation (<0.65 nm). While the out-of-plane polarizability of WS_2_ measured at room temperature shows a larger value,^39^ we note that thermal energy at high temperature may impact the binding energy of the exciton, increasing the effective Bohr radius. As a result, the *X* ^0^ exciton in WSe_2_ is more localized at low temperature, leading to suppressed polarizability.

**Table 1:** Out-of-Plane Polarizability (α_⊥_) of the *X* ^0^ Exciton in TMDs Obtained from Experiments

Material	Dielectric^a^	Temperature^b^	α_⊥_ (D·mV^–1^)
WSe_2_	h-BN	4 K	≤10^–11^ (ref 25)
WS_2_	h-BN	Room Temperature	∼1.1 × 10^–9^ (ref 39)
MoS_2_	h-BN	4 K	(7.8 ± 1.0) × 10^–10^ (ref 38)
MoS_2_	SiO_2_ and Al_2_O_3_	10 K	(2.9 ± 1.25)× 10^–9^ (ref 37)

a Dielectric materials affect the binding energy of the exciton.

b Temperature affects the linewidth, thus influencing the resolution of the measurements.

Interestingly, our measurements show that the out-of-plane polarizability of the *D* ^0^ exciton is <6.7 × 10^–10^ D·mV^–1^, which indicates the *D* ^0^ exciton is stiffer than the in-plane oscillating *X* ^0^ exciton in MoS_2_ and WS_2_. This suggests that excitons are more tightly bound in WSe_2_ compared with other TMD compounds. While the oscillating dipoles of *X* ^0^ and *D* ^0^ are mutually orthogonal, their out-of-plane polarizabilities remain comparably small (both our measurements and those by Verzhbitskiy et al.^25^ are limited by the equipment resolution). Although *X* ^0^ and *D* ^0^ excitons are distinct in the spin state and dipole orientation, they share the same geometric confinement within the monolayer. Despite the fact that Coulomb interaction is nonlocal,^40^ the electric field perturbation is negligible for both species. Compared to the neutral *D* ^0^ exciton, the experimental investigations on the charged *D* ^+^ and *D* ^–^ excitons are more complicated, as both shift with increasing carrier concentration. We show the discussion on the charged excitons and phonon replica peak in Figures S7–S9 and focus on the *D* ^0^ peak in the main text.

Although the minimal out-of-plane dipole is theoretically expected due to the monolayer's thickness and mirror symmetry, the *D* ^0^ exciton provides a distinct functional advantage over interlayer excitons. Contrary to the interlayer excitons that are electric field-tunable,^15–18^ being insensitive to electric fields means that the dark excitons are robust against charge noise. Our results suggest that the dark exciton in WSe_2_ has significant potential as a robust coherent two-level system protected against the primary dephasing mechanism in solid-state devices.

To further examine the charge noise resilience, we performed magneto-optical measurements at low and high electric fields. Figure 5a,b shows the splitting of *D* ^0^ exciton at −0.1 and 1.3 MV/cm, respectively. Thanks to the narrow linewidth, we are able to identify fine structure splitting of the *D* ^0^ exciton, which exhibits a parabolic pattern as a function of the magnetic field (*B*) (See Figure S10 for Device 1). Our observation is consistent with Robert et al.,^19^ with the low-energy state being strictly dark at *B* = 0 T. In Figure 5c,d, we plotted the *B*-dependent splitting energy, Δ*E*(*B*), by calculating the difference in peak positions between the high-energy and low-energy states. To extract the *g*-factor, we fitted the experimental data by the following equation

$$\Delta E\left(B\right)=\sqrt{{\left(g\mu_{B}B\right)}^{2}+\delta^{2}}$$

where μ_B_ = 58 μ*eV*/*T* is the Bohr magneton and δ is the zero-field splitting energy. Due to intrinsic optical resolution limits, the small splitting at low magnetic fields (|*B*| < 2 T) cannot be clearly resolved. Consequently, leaving δ as a free parameter during the fitting procedure yields a value with considerable uncertainty (δ = 0.38 ± 0.47; see Figure S11). To ensure a robust and physically meaningful fit, we fixed δ = 0.6 meV. This constrained value is strongly supported by our independent measurements on a separate device (δ = 0.57 meV, Figure S10) and is highly consistent with that in the literature.^19^ A full comparison of the fixed- and free-parameter fitting procedures is detailed in Figures S10–S11.

**Figure 5:**
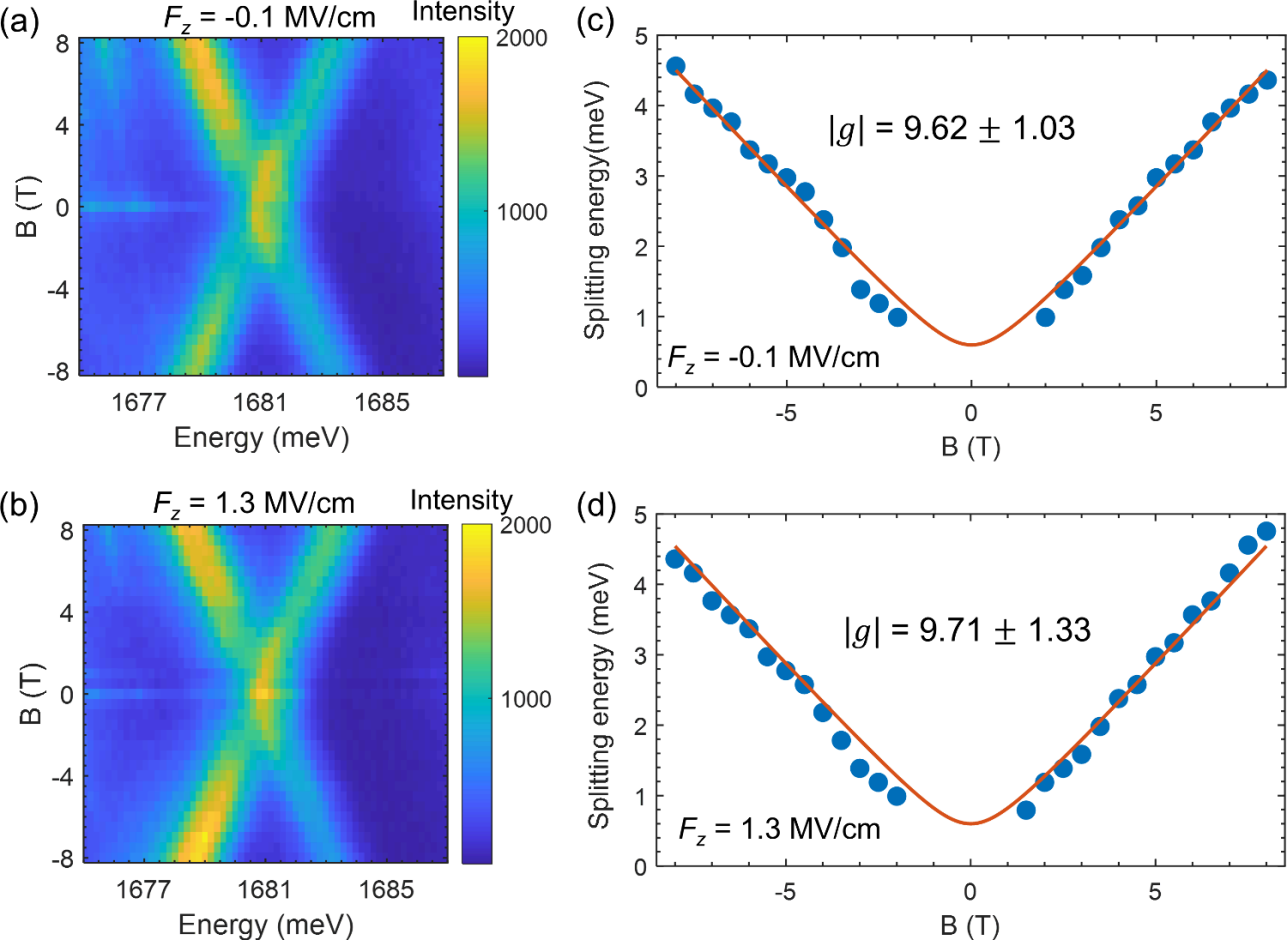
Magnetic field dependence of *D* ^0^ exciton in Device 4. (a, b) PL color plots of the *D* ^0^ exciton as a function of *B* at −0.1 MV/cm (a) and 1.3 MV/cm (b). (c, d) *B*-dependent splitting energy of the dark exciton state at −0.1 MV/cm (c) and 1.3 MV/cm (d). Experimental data are shown by the blue dots, and fits are indicated by the red lines. Error bars of the *g*-factors are derived from the fitting procedure. The absence of experimental data at low *B* is due to the unresolved low-energy state, which is brightened at higher *B*.

We obtained |*g*| = 9.62 ± 1.03 and |*g*| = 9.71 ± 1.33 for the low and high electric fields, respectively. Our measurements indicate that the magnetic moment of *D* ^0^ exciton is decoupled from the out-of-plane electric field, different from the highly tunable interlayer exciton.^41^ Additionally, our result is also in contrast to the carrier-dependent splitting of dark charged excitons at a finite *B*, where the interaction between the dark exciton-polaron and dark trion alters the *g*-factor.^42^

In summary, we presented an experimental study of the electric field dependence of the spin-triplet dark exciton in dual-gated monolayer WSe_2_. Despite oscillating in the same direction as the electric field, the *D* ^0^ exciton shows a small out-of-plane polarizability with an upper limit of 6.7 × 10^–10^ D·mV^–1^, which is even lower than that of the in-plane *X* ^0^ exciton in WS_2_ and MoS_2_. We attribute our measured polarizability to the strong spatial confinement of the monolayer sample; conversely, the larger polarizability from WS_2_ and MoS_2_ could be explained by the leakage of the wave function into the barriers. While the tunability from interlayer exciton is a desirable feature, we note that insensitivity to the electrical perturbations is equally valuable, particularly in quantum science and technology. Beyond testing the theoretical models on excitons, we demonstrate that the *g*-factor from the *D* ^0^ exciton is also robust against charge noise. This stability against electrical perturbations positions the dark excitonic states as a critical, noise-resilient constituent for future spin–valley quantum architectures. Our findings not only clarify the electric field response of the dark excitons but also provide a powerful blueprint for utilizing these long-lived states as a foundational building block, paving the way to exploit the intrinsic stiffness of the monolayer environment for enhanced quantum coherence.

## Supplementary Material



## Notes

Methods: Device fabrication, Optical spectroscopy measurements, and Back-focal-plane (BFP) imaging; Schematic diagram of the back-focal-plane imaging measurements; PL color plot from Device 1; Thickness of the h-BN layers; Electric field dependence of *X* ^0^ and *D* ^0^ excitons in Device 1 measured in the second cool-down cycle; PL spectra of *X* ^0^ and *D* ^0^ excitons at different electric fields in Device 3; Electric field dependence of *X* ^0^ and *D* ^0^ excitons in Device 4; Optical microscopy images of Device 3 and Device 4; Gate-dependent shift of the charged *D* ^+^ and *D* ^–^ excitons; *V*_BG_-dependent shift of the *D* ^–^ exciton at *V*_TG_ = 0 V and *V*_TG_ = +3 V; PL spectra of the *D*_Γ_5__^0^ phonon replica peak at low and high electric fields; Magnetic field dependence of *D* ^0^ exciton in Device 1; *B*-dependent splitting energy of the *D* ^0^ exciton at *F*_*z*_ = −0.1 MV/cm (PDF)

## ☆

Corning Incorporated, Corning, New York, United States

## References

[1] Wang G., Chernikov A., Glazov M. M., Heinz T. F., Marie X., Amand T., Urbaszek B. (2018). Colloquium: Excitons in atomically
thin transition metal dichalcogenides. Rev.
Mod. Phys..

[2] Xu X., Yao W., Xiao D., Heinz T. F. (2014). Spin and pseudospins
in layered transition
metal dichalcogenides. Nat. Phys..

[3] Aslan B., Deng M., Heinz T. F. (2018). Strain
tuning of excitons in monolayer
WSe_2_. Phys. Rev. B.

[4] Niehues I., Schmidt R., Druppel M., Marauhn P., Christiansen D., Selig M., Berghäuser G., Wigger D., Schneider R., Braasch L. (2018). Strain control of exciton–phonon
coupling in
atomically thin semiconductors. Nano Lett..

[5] Raja A., Chaves A., Yu J., Arefe G., Hill H. M., Rigosi A. F., Berkelbach T. C., Nagler P., Schüller C., Korn T. (2017). Coulomb engineering
of the bandgap and excitons in two-dimensional
materials. Nat. Commun..

[6] Van
Tuan D., Yang M., Dery H. (2018). Coulomb interaction in monolayer
transition-metal dichalcogenides. Phys. Rev.
B.

[7] Mak K. F., He K., Lee C., Lee G. H., Hone J., Heinz T. F., Shan J. (2013). Tightly bound trions
in monolayer MoS_2_. Nat. Mater..

[8] Ross J. S., Wu S., Yu H., Ghimire N. J., Jones A. M., Aivazian G., Yan J., Mandrus D. G., Xiao D., Yao W. (2013). Electrical control
of neutral and charged excitons in a monolayer semiconductor. Nat. Commun..

[9] Zhu B., Xiao K., Yang S., Watanabe K., Taniguchi T., Cui X. (2023). In-plane electric-field-induced orbital hybridization of excitonic
states in monolayer WSe_2_. Phys. Rev.
Lett..

[10] Massicotte M., Vialla F., Schmidt P., Lundeberg M. B., Latini S., Haastrup S., Danovich M., Davydovskaya D., Watanabe K., Taniguchi T. (2018). Dissociation
of two-dimensional excitons
in monolayer WSe_2_. Nat. Commun..

[11] Lian Z., Li Y.-M., Yan L., Ma L., Chen D., Taniguchi T., Watanabe K., Zhang C., Shi S.-F. (2024). Stark Effects
of Rydberg Excitons in a Monolayer WSe_2_ P–N Junction. Nano Lett..

[12] Mak K. F., Shan J. (2016). Photonics and optoelectronics
of 2D semiconductor transition metal
dichalcogenides. Nat. Photonics.

[13] Wu J., Ma H., Yin P., Ge Y., Zhang Y., Li L., Zhang H., Lin H. (2021). Two-dimensional
materials for integrated
photonics: recent advances and future challenges. Small Sci..

[14] Liu X., Hersam M. C. (2019). 2D materials for quantum information science. Nat. Rev. Mater..

[15] Ciarrocchi A., Unuchek D., Avsar A., Watanabe K., Taniguchi T., Kis A. (2019). Polarization switching
and electrical control of interlayer excitons
in two-dimensional van der Waals heterostructures. Nat. Photonics.

[16] Peimyoo N., Deilmann T., Withers F., Escolar J., Nutting D., Taniguchi T., Watanabe K., Taghizadeh A., Craciun M. F., Thygesen K. S. (2021). Electrical
tuning of optically active
interlayer excitons in bilayer MoS_2_. Nat. Nanotechnol..

[17] Leisgang N., Shree S., Paradisanos I., Sponfeldner L., Robert C., Lagarde D., Balocchi A., Watanabe K., Taniguchi T., Marie X. (2020). Giant Stark splitting
of an exciton
in bilayer MoS_2_. Nat. Nanotechnol..

[18] Jauregui L. A., Joe A. Y., Pistunova K., Wild D. S., High A. A., Zhou Y., Scuri G., De Greve K., Sushko A., Yu C.-H. (2019). Electrical control of interlayer exciton dynamics in atomically thin
heterostructures. Science.

[19] Robert C., Amand T., Cadiz F., Lagarde D., Courtade E., Manca M., Taniguchi T., Watanabe K., Urbaszek B., Marie X. (2017). Fine structure and
lifetime of dark excitons in transition metal
dichalcogenide monolayers. Phys. Rev. B.

[20] Tang Y., Mak K. F., Shan J. (2019). Long valley lifetime of dark excitons
in single-layer WSe_2_. Nat. Commun..

[21] He M., Rivera P., Van Tuan D., Wilson N. P., Yang M., Taniguchi T., Watanabe K., Yan J., Mandrus D. G., Yu H. (2020). Valley phonons
and exciton complexes in a monolayer semiconductor. Nat. Commun..

[22] Liu E., van Baren J., Taniguchi T., Watanabe K., Chang Y.-C., Lui C. H. (2019). Valley-selective chiral phonon replicas of dark excitons
and trions in monolayer WSe_2_. Phys.
Rev. Research.

[23] Li Z., Wang T., Jin C., Lu Z., Lian Z., Meng Y., Blei M., Gao S., Taniguchi T., Watanabe K. (2019). Emerging photoluminescence from the
dark-exciton phonon
replica in monolayer WSe_2_. Nat. Commun..

[24] Yang M., Ren L., Robert C., Van Tuan D., Lombez L., Urbaszek B., Marie X., Dery H. (2022). Relaxation and darkening of excitonic
complexes in electrostatically doped monolayer WSe_2_: Roles
of exciton-electron and trion-electron interactions. Phys. Rev. B.

[25] Verzhbitskiy I., Vella D., Watanabe K., Taniguchi T., Eda G. (2019). Suppressed out-of-plane polarizability
of free excitons in monolayer
WSe_2_. ACS Nano.

[26] Zhang X.-X., Cao T., Lu Z., Lin Y.-C., Zhang F., Wang Y., Li Z., Hone J. C., Robinson J. A., Smirnov D. (2017). Magnetic brightening
and control of dark excitons in monolayer WSe_2_. Nat. Nanotechnol..

[27] Zhou Y., Scuri G., Wild D. S., High A. A., Dibos A., Jauregui L. A., Shu C., De Greve K., Pistunova K., Joe A. Y. (2017). Probing dark excitons
in atomically thin semiconductors
via near-field coupling to surface plasmon polaritons. Nat. Nanotechnol..

[28] Chen P., Cheng C., Shen C., Zhang J., Wu S., Lu X., Wang S., Du L., Watanabe K., Taniguchi T. (2019). Band evolution
of two-dimensional transition metal dichalcogenides under electric
fields. Appl. Phys. Lett..

[29] Brotons-Gisbert M., Proux R., Picard R., Andres-Penares D., Branny A., Molina-Sánchez A., Sánchez-Royo J. F., Gerardot B. D. (2019). Out-of-plane orientation
of luminescent excitons in
two-dimensional indium selenide. Nat. Commun..

[30] Robert C., Park S., Cadiz F., Lombez L., Ren L., Tornatzky H., Rowe A., Paget D., Sirotti F., Yang M. (2021). Spin/valley
pumping of resident electrons in WSe_2_ and
WS_2_ monolayers. Nat. Commun..

[31] Jiang C., Rasmita A., Xu W., Imamoğlu A., Xiong Q., Gao W.-b. (2018). Optical spin pumping
induced pseudomagnetic
field in two-dimensional heterostructures. Phys.
Rev. B.

[32] Li W., Lu X., Wu J., Srivastava A. (2021). Optical control
of the valley Zeeman
effect through many-exciton interactions. Nat.
Nanotechnol..

[33] Cadiz F., Courtade E., Robert C., Wang G., Shen Y., Cai H., Taniguchi T., Watanabe K., Carrere H., Lagarde D. (2017). Excitonic
linewidth approaching the homogeneous limit in MoS _2_-based
van der Waals heterostructures. Phys. Rev. X.

[34] Kim K., Larentis S., Fallahazad B., Lee K., Xue J., Dillen D. C., Corbet C. M., Tutuc E. (2015). Band alignment
in WSe_2_–graphene heterostructures. ACS
Nano.

[35] Laturia A., Van de Put M. L., Vandenberghe W. G. (2018). Dielectric properties of hexagonal
boron nitride and transition metal dichalcogenides: from monolayer
to bulk. npj 2D Mater. Appl..

[36] Pedersen T. G. (2016). Exciton
Stark shift and electroabsorption in monolayer transition-metal dichalcogenides. Phys. Rev. B.

[37] Klein J., Wierzbowski J., Regler A., Becker J., Heimbach F., Muller K., Kaniber M., Finley J. J. (2016). Stark effect
spectroscopy
of mono-and few-layer MoS_2_. Nano
Lett..

[38] Roch J. G., Leisgang N., Froehlicher G., Makk P., Watanabe K., Taniguchi T., Schonenberger C., Warburton R. J. (2018). Quantum-confined
Stark effect in a MoS_2_ monolayer van der Waals heterostructure. Nano Lett..

[39] Abraham N., Watanabe K., Taniguchi T., Majumdar K. (2021). Anomalous Stark shift
of excitonic complexes in monolayer WS_2_. Phys. Rev. B.

[40] Chernikov A., Berkelbach T. C., Hill H. M., Rigosi A., Li Y., Aslan B., Reichman D. R., Hybertsen M. S., Heinz T. F. (2014). Exciton binding
energy and nonhydrogenic Rydberg series
in monolayer WS_2_. Phys. Rev. Lett..

[41] Feng S. (2024). Highly tunable
ground and excited state excitonic dipoles in multilayer 2H-MoSe_2_. Nat. Commun..

[42] Cong X., Mohammadi P. A., Zheng M., Watanabe K., Taniguchi T., Rhodes D., Zhang X.-X. (2023). Interplay of valley
polarized dark
trion and dark exciton-polaron in monolayer WSe_2_. Nat. Commun..

