# Supplementary material for: Vanishing Polarizability of Dark Excitons in WSe2: Implications for Noise-Resilient Quantum States
Source: Nano Lett. 2026 Apr 27;26(17):5945–51. doi: 10.1021/acs.nanolett.6c01047 (PMC13154348; doi:10.1021/acs.nanolett.6c01047)
Supplement: Supplementary file 1 [file nl6c01047_si_001.pdf]

Supporting Information

Vanishing Polarizability of Dark Excitons in WSe_2: Implications for Noise-Resilient Quantum States

Ali Soleymani[1], Qiaohui Zhou[1†], Keyuan Bai[1], Fei Wang[1], Kenji Watanabe[2], Takashi Taniguchi[3], Jiang Wei[1], and Xin Lu[1]*

[1]Department of Physics and Engineering Physics,

Tulane University, New Orleans, Louisiana 70118, United States

[2]Research Center for Electronic and Optical Materials,

National Institute for Materials Science, 1-1 Namiki, Tsukuba 305-0044, Japan and

[3]Research Center for Materials Nanoarchitectonics,

National Institute for Materials Science, 1-1 Namiki, Tsukuba 305-0044, Japan.

*Xin Lu. Email: xlu5@tulane.edu.

Present Addresses

[†]Qiaohui Zhou: Corning Incorporated, Corning, New York, United States

Methods

Device fabrication. Electron beam lithography was used to define 5 nm Ti/100 nm Au metal electrodes on the SiO_2/Si substrate. Separately exfoliated flakes of monolayer WSe_2 (HQ Graphene), few-layer graphene, and h-BN were then sequentially assembled onto the substrate by using a polydimethylsiloxane (PDMS)-based dry transfer technique.[1] The WSe_2 monolayer is sandwiched between the two h-BN flakes, which not only protect the channel but also serve as gate dielectrics. Few-layer graphene was used to form both the bottom and top gate electrodes, where the bottom-gate (V_{BG}) and top-gate (V_{TG}) voltages are applied, respectively. Another few-layer graphene flake was transferred to act as the grounding contact.

Optical spectroscopy measurements. To conduct low-temperature magneto-optical characterization, the samples were mounted in a closed-cycle cryostat (AttoDry 1000, Attocube Systems) featuring a superconducting magnet. We utilized piezoelectric nanopositioners (Attocube Systems) to align the sample at a base temperature of ~3.7 K. Excitation was provided by a 633-nm continuous-wave laser, focused to a ~1 μm spot size via a low-temperature objective (NA = 0.81, Attocube Systems). Excitation power ranged from 5 μW to 20 μW, depending on the sample. The resulting emission was collected through the same objective and analyzed by using a high-resolution spectrometer (HRS-750, Teledyne Princeton Instruments) equipped with a liquid nitrogen-cooled detector (PYL-400BRX, Teledyne Princeton Instruments). Polarization was achieved using a λ/4 waveplate positioned after the beamsplitter, and a λ/2 waveplate, followed by a fixed-polarization analyzer, was placed at the spectrometer entrance.

Back-focal-plane (BFP) imaging. We follow References 2 and 3 to set up the measurements. In Figure S1, Lens 1 and Lens 2 (Thorlabs) serve as relay lenses, mapping the back focal plane of the low-temperature objective onto the pinhole. At this plane, we collected the angle (θ)-dependent emission. To cover full emission range of

$\pm 54°$ (determined by the NA of the objective), the pinhole is mounted on a motorized translation stage controlled via computer script.

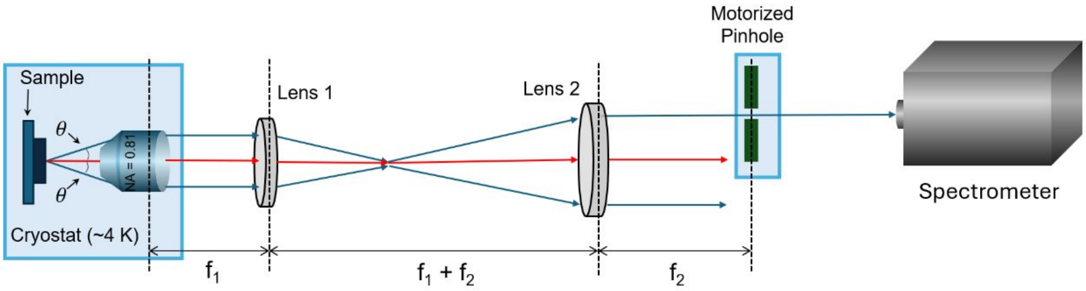

Figure S1. Schematic diagram of the back-focal-plane imaging measurements. Lens 1 and Lens 2 (Thorlabs) serve as relay lenses, mapping the back focal plane of the low-temperature objective onto the pinhole. At this plane, we collected the angle (θ)-dependent emission. To cover full emission range of $\pm 54°$ (determined by the NA of the objective), the pinhole is mounted on a motorized translation stage controlled via computer script. The relative positions among the low-temperature objective, Lens 1, Lens 2, and the pinhole are determined by the focal lengths of relay lenses. For simplicity, beamsplitter, long-pass filter, and lens before the spectrometer are not shown in the figure.

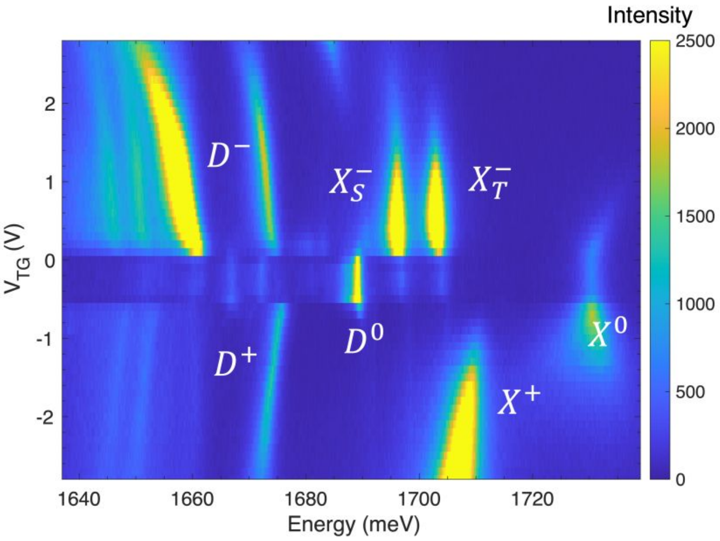

Figure S2. PL color plot as a function V_{TG}. The measurements were performed with $V_{BG} = 0$ V from Device 1. The excitation laser is linearly polarized.

Table S1. Thicknesses of the h-BN layers in Devices 1, 3, and 4.

Device	Top h-BN	Bottom h-BN
**Device 1**	27 nm	22 nm
**Device 3**	18 nm	18 nm
**Device 4**	17 nm	19 nm

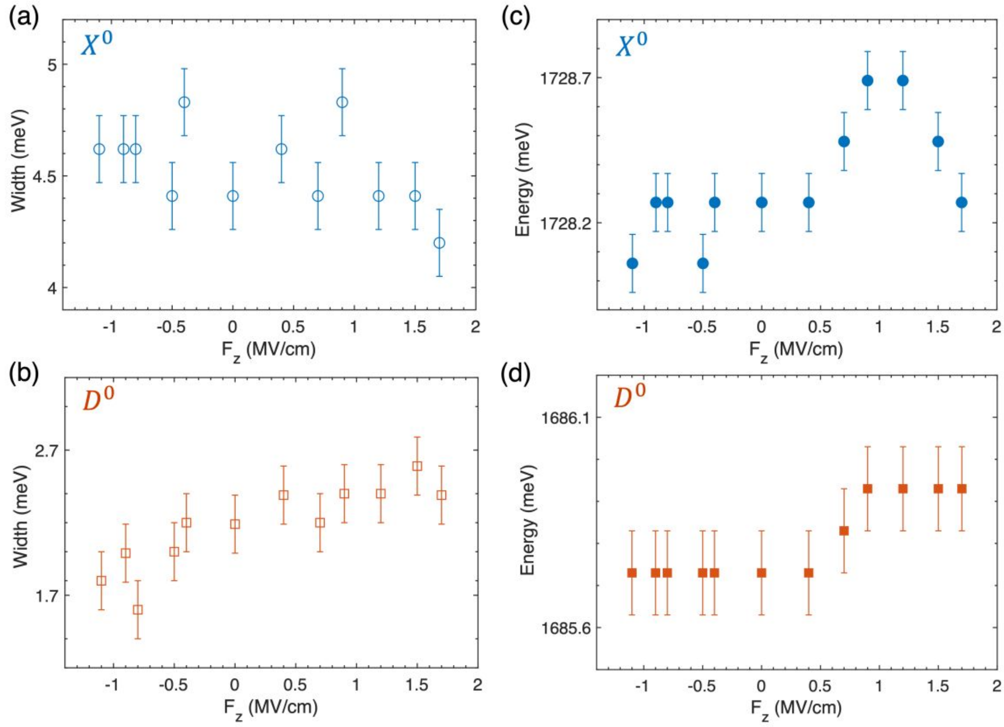

Figure S3. Electric field-dependence of X^0 and D^0 excitons in Device 1 measured in the second cool-down cycle. (a, b) F_z-dependent peak width of X^0(a) and D^0(b). (c, d) F_z-dependent peak energy of X^0(c) and D^0(d).

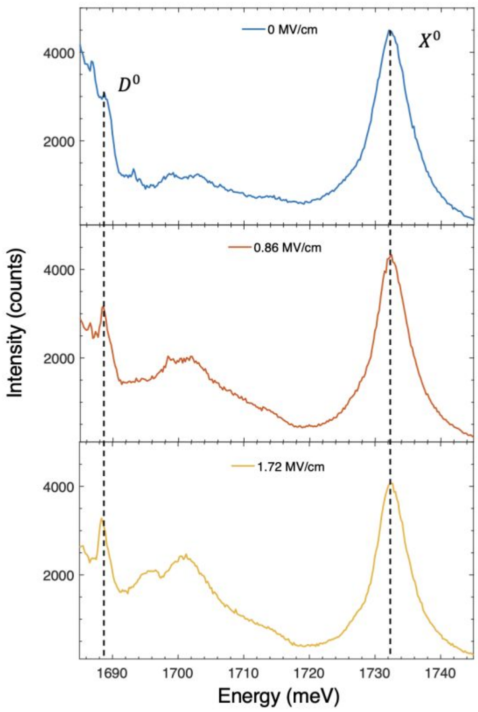

Figure S4. PL spectra of X^0 and D^0 excitonic peaks at 0, 0.86, and 1.72 MV/cm in Device 3.

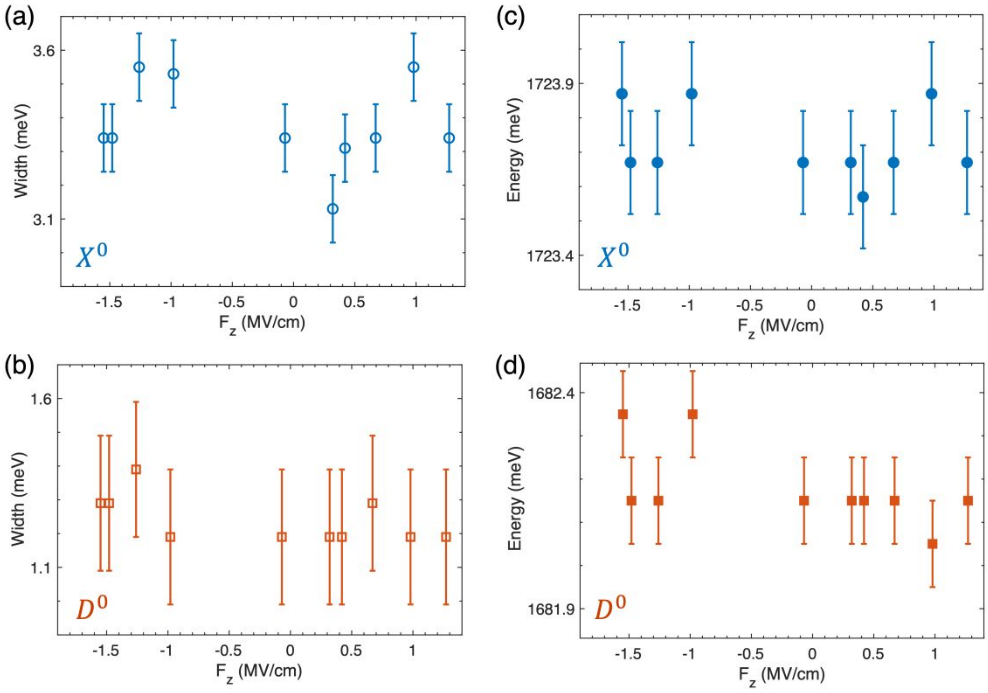

Figure S5. Electric field-dependence of X^0 and D^0 excitons in Device 4. (a, b) F_z-dependent peak width of X^0(a) and D^0(b). (c, d) F_z-dependent peak energy of X^0(c) and D^0(d).

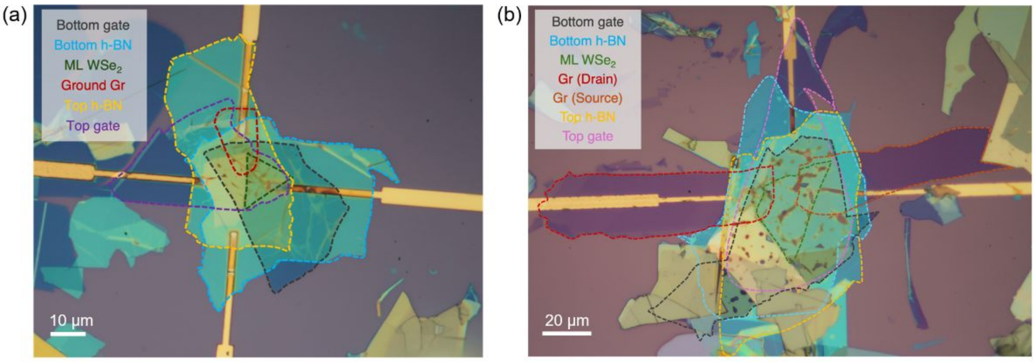

Figure S6. Optical microscopy images of Device 3 (a) and Device 4 (b).

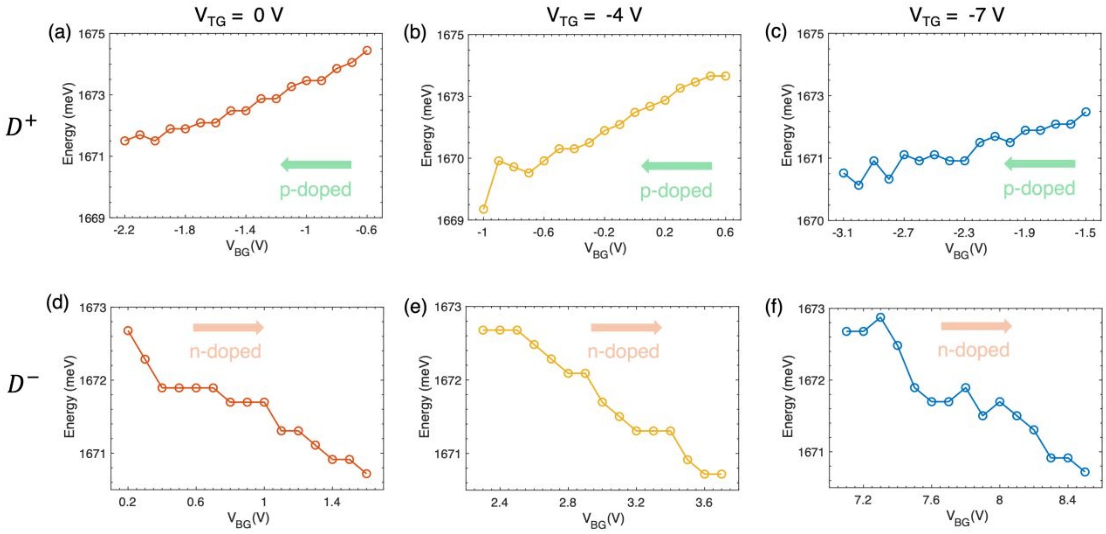

Figure S7. Gate-dependent shift of the charged D^+ and D^- excitons. (a-c) V_{BG}-dependent shift of the D^+ exciton at V_{TG} = 0 V (a), V_{TG} = -4 V(b), and V_{TG} = -7 V(c). (d-f) V_{BG}-dependent shift of the D^- exciton at V_{TG} = 0 V (d), V_{TG} = -4 V(e), and V_{TG} = -7 V(f). Data for V_{TG} = 0 V, V_{TG} = -4 V, and V_{TG} = -7 V are shown in red, yellow, and blue respectively. The thick arrows indicate the direction of increasing concentration in the p-doped or n-doped regions. Although both D^+ and D^- excitons red-shift with increasing doping density, it is evident that the overall energy of the D^+ exciton decreases with the electric field (a-c) while the D^- peak exhibits a negligible shift as V_{TG} changes (d-f). We attribute the unexpected shift of the D^+ exciton to the asymmetric dielectric environment, as the WSe_2 monolayer is not symmetrically encapsulated; the top and bottom h-BN layers are not from the same flake (tear-and-stack), nor are they of the same thickness. To test our hypothesis, we reversed the sign of V_{TG} and performed the same measurements in Figure S8.

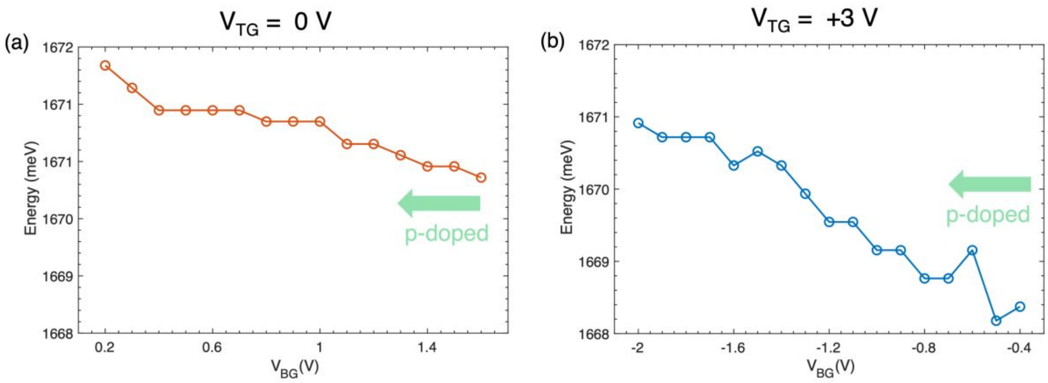

Figure S8. V_{BG}-dependent shift of the D^- exciton at V_{TG} = 0 V (a) and V_{TG} = +3 V(b). While the D^- exciton is insensitive to the electric field when $V_{TG} \leq 0$ V (Figure S7d-f), the negatively charged peak exhibits a substantial redshift when V_{TG} = +3 V. Our observations point to an asymmetric dielectric environment effect. As the charged peaks carry a net charge, they would be pushed toward one of the interfaces (either the top or bottom) when the out-of-plane electric field is nonzero. The fact that D^+ (D^-) redshifts when $V_{TG} \leq 0$ ($V_{TG} \geq 0$) indicates that the top WSe_2/h-BN interface can cause stronger local dielectric screening effect, thereby lowering the energy. Note that since D^0 is charge-neutral, it is not affected by the asymmetric dielectric environment.

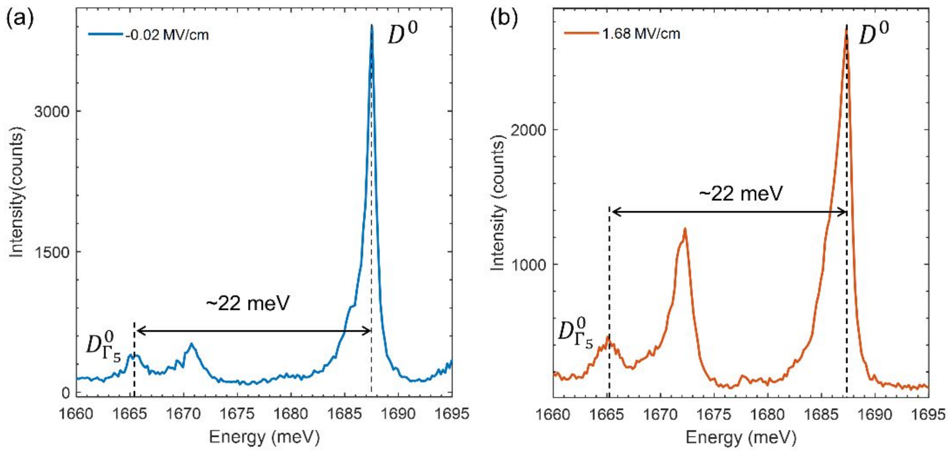

Figure S9. PL spectra of the $D^0_{\Gamma_5}$ phonon replica peak at low (a) and high (b) electric fields, showing no observable shift. Since the energy of a phonon is generally insensitive to electric fields, we do not expect that the $D^0_{\Gamma_5}$ peak to show a measurable shift as the field increases from -0.02 MV/cm to 1.68 MV/cm.

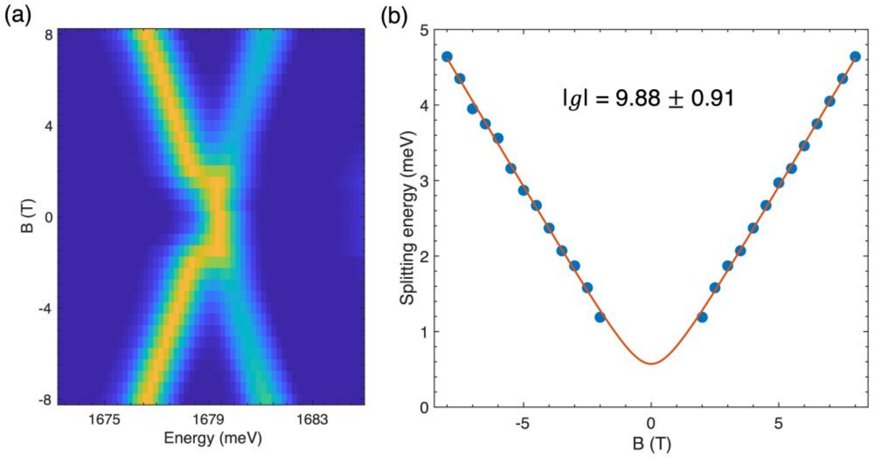

Figure S10. Magnetic field dependence of D^0 exciton in Device 1. (a) Normalized intensity map of the D^0 exciton as a function of B. (b) B-dependent splitting energy of the dark exciton state. Experimental data are shown by the blue dots, and fit is indicated by the red line. Fitting was performed without fixing the zero field splitting energy (δ). Consistent with an earlier report,[1] we obtained $\delta = 0.57\,meV$ from the linear fit. Error bar of the g-factor is derived from the fitting procedure. The absence of experimental data at low B is due to the unresolved low-energy state, which is brightened at higher B.

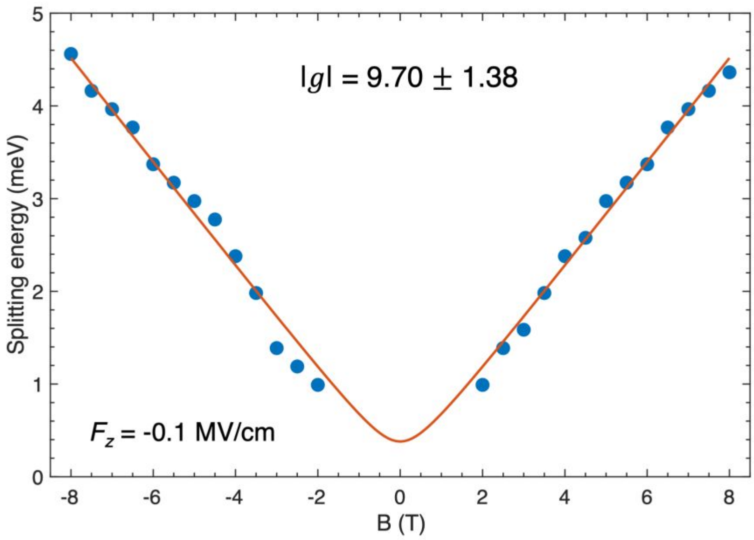

Figure S11. B-dependent splitting energy of the D^0 exciton at F_z = -0.1 MV/cm in Device 4. Experimental data are shown by the blue dots, and fit is indicated by the red line. The fitting was performed without fixing the zero-field splitting (δ). We obtained δ = 0.38 $\pm$ 0.47 and $|g|$ = 9.70 $\pm$ 1.38 from the fitting procedure. Note that the fitting error for δ is large, indicating δ could range from 0 to 0.85 meV. In Figure 5 of the main text, we fixed $\delta = 0.6\,meV$, which is in accordance with results from the literature[4] and Device 1 (Figure S10).

Reference

(1) Castellanos-Gomez, A.; Buscema, M.; Molenaar, R.; Singh, V.; Janssen, L.; Van Der Zant, H. S.; Steele, G. A. Deterministic transfer of two-dimensional materials by all-dry viscoelastic stamping. *2D Mater.* **2014**, *1* (1), 011002.

(2) Luo, Y.; Liu, N.; Kim, B.; Hone, J.; Strauf, S. Exciton dipole orientation of strain-induced quantum emitters in WSe_2. *Nano Lett.* **2020**, *20* (7), 5119-5126.

(3) Kumar, P. Imaging and spectroscopy of solid-state quantum emitters. Ph.D. Thesis, Heriot-Watt University, Edinburgh, UK, 2018.

(4) Robert, C.; Amand, T.; Cadiz, F.; Lagarde, D.; Courtade, E.; Manca, M.; Taniguchi, T.; Watanabe, K.; Urbaszek, B.; Marie, X. Fine structure and lifetime of dark excitons in transition metal dichalcogenide monolayers. *Phys. Rev. B* **2017**, *96* (15), 155423.
